# Cognitive Symptoms Link Anxiety and Depression Within a Validation of the German State-Trait Inventory for Cognitive and Somatic Anxiety (STICSA)

**DOI:** 10.32872/cpe.9753

**Published:** 2023-06-29

**Authors:** Rebecca Overmeyer, Tanja Endrass

**Affiliations:** 1 Faculty of Psychology, Institute of Clinical Psychology and Psychotherapy, Chair for Addiction Research, Technische Universität Dresden, Dresden, Germany; Philipps-University of Marburg, Marburg, Germany

**Keywords:** questionnaire, anxiety, depression, somatic symptoms, cognitive symptoms

## Abstract

**Background:**

In the present study we aimed to develop a German version of the State-Trait Inventory for Cognitive and Somatic Anxiety (STICSA) and evaluate the psychometric properties. Associations of cognitive and somatic anxiety with other measures of anxiety, depression, and stress, elucidating possible underlying functional connections, were also examined, as symptoms of anxiety, depression and stress often overlap.

**Method:**

Two samples (n1 = 301; n2 = 303) were collected online and in the lab, respectively. Dynamic connections between somatic and cognitive anxiety, other measures of anxiety, depression, and stress, were analyzed using a network approach. Psychometric analyses were conducted using exploratory and confirmatory factor analyses.

**Results:**

We replicated and validated the two-factorial structure of the STICSA with the German translation. Network analyses revealed cognitive trait anxiety as the most central node, bridging anxiety and depression. Somatic trait anxiety exhibited the highest discriminant validity for distinguishing anxiety from depression.

**Conclusion:**

The central role of cognitive symptoms in these dynamic interactions suggests an overlap of these symptoms between anxiety and depression and that differential diagnostics should focus more on anxious somatic symptoms than on cognitive symptoms. The STICSA could therefore be useful in delineating differences between anxiety and depression and for differential assessment of mood and anxiety symptoms. Additional understanding of both cognitive and somatic aspects of anxiety might prove useful for therapeutic interventions.

## Highlights

- Cognitive symptoms link depression and anxiety within a network approach.
- Somatic symptoms exhibit high discriminant validity towards depression.
- Differentiating subcomponents of anxious symptoms may help differentiate anxiety and depression.
- The German version of the STICSA is a reliable and valid measure of trait anxiety.

Anxiety disorders and depression are among the most prevalent mental disorders, are highly comorbid and cause a high burden of disease (Bandelow & Michaelis, 2015; Leray et al., 2011; Martin, 2003; Michael et al., 2007). Symptoms of anxiety, depression and stress often overlap (Mineka et al., 1998) and identifying overlapping and distinctive features of anxiety and depression is highly important (Eysenck & Fajkowska, 2018). Anxiety and depression are clearly not identical emotional states, but the high comorbidity rate and the diagnostic overlap point to common nonspecific features and mechanisms, that are also important for treatment (Eysenck & Fajkowska, 2018; Marchetti et al., 2016). There is also evidence that anxiety and depression dynamically interact and may trigger each other (Starr & Davila, 2012a, 2012c).

Anxiety can be divided into state and trait anxiety (e.g. Endler & Kocovski, 2001). Trait anxiety is a stable predisposition to experience anxiousness or to experience state anxiety frequently (Spielberger, 1966). State anxiety is an anxiety experienced within a specific moment and varies significantly between individuals and is associated with the development of pathological anxiety when experienced more often and with high intensity (Spielberger, 1966). Many models describing anxiety emphasize the multidimensionality of anxiety. This is particularly important when aiming for comprehensive assessment of anxiety and distinguishing anxiety from depression. Dimensions include cognitive, physiological and behavioral aspects of anxiety (Elwood et al., 2012). So far, established measures of anxiety rarely distinguish between cognitive and somatic dimensions of anxiety. The Cognitive Somatic Anxiety Questionnaire (Delmonte & Ryan, 1983; Schwartz et al., 1978) and the Endler Multidimensional Anxiety Scales (Endler et al., 1991) both include scales on cognitive and somatic symptoms but exclusively focus on trait assessment.

Distinguishing between anxiety and depression requires examining the complex and multilayered facets of both syndromes (Eysenck & Fajkowska, 2018). Several approaches examine anxiety and depression in a common theoretical framework. One approach suggests that anxiety focuses on the future and depression on the past resulting in respective cognitive biases (Eysenck et al., 2006; Pomerantz & Rose, 2014). However, there is evidence that worry and rumination differ in their effects on behavioral and physiological responses to every day events and stressors, and that there is not a specific link between anxiety and worry, or depression and rumination (Kircanski et al., 2017; Lewis et al., 2018). Beck’s content-specificity hypothesis suggests that anxiety is marked by a focus on danger, and in depression by self-deprecation (Beck, 1976; Beck et al., 1987). Lastly, the tripartite model of anxiety and depression posits that anxiety and depression share a component of underlying negative affectivity or distress but anxiety is additionally marked by physiological hyperarousal, whereas depression is additionally marked by low positive affectivity (Clark, 2009; Clark & Watson, 1991). However, none of these approaches can fully capture the complexity of how anxiety and depression overlap, how they differ, and how they interact (Eysenck & Fajkowska, 2018).

In addition, some of the established instruments for the assessment of anxiety exhibit low discriminant validity regarding depressive symptoms. For instance, the State-Trait Anxiety Inventory (STAI; Spielberger et al., 1983) is almost exclusively used to assess state and trait anxiety, but recent findings suggest that the STAI also assesses depressive symptoms alongside anxiety. Anxiety and depressive symptom severity are similarly correlated with the STAI trait and state score, and individuals with depressive disorders score significantly higher on average than individuals with anxiety disorders (Kennedy et al., 2001; Knowles & Olatunji, 2020). Both anxiety and depression appear to share a component of negative affect (e.g. Anderson & Hope, 2008; Balon, 2005; Bieling et al., 1998; Caci et al., 2003).

In clinical research and practice, it is important to assess distinct aspects of anxiety, rather than just negative affectivity. Therefore, an instrument is needed that validly assesses anxiety, separately from depressive symptoms. In contrast to other questionnaires, the State-Trait Inventory for Cognitive and Somatic Anxiety (STICSA; Ree et al., 2008) aims to measure anxiety without including negative affectivity. The STICSA has 21 items for the state and trait scales, respectively, and has been shown to be a reliable instrument for the assessment of anxiety. The STICSA considers the multidimensionality of anxiety, as well as the need to differentiate it from depressive symptoms (Elwood et al., 2012; Grös et al., 2007; Ree et al., 2008). While the two-factorial structure of cognitive and somatic anxiety has been validated for the state and trait scale of the STICSA, other factorial solutions have also been proposed. Factor solutions for all items of the STICSA state and trait version revealed a four-factor model, as well as a higher-order model with a global anxiety factor and four first-order factors (STICSA trait cognitive subscale, STICSA trait somatic subscale, STICSA state cognitive subscale, and STICSA state somatic subscale). Aside from the two-factor solutions for the trait and state scale, respectively, utilized by Ree et al. (2008), these four-factor solutions have also been validated (Carlucci et al., 2018; Roberts et al., 2016). Superior concurrent and divergent validity has been shown compared to the STAI (Tindall et al., 2021). So far, the STICSA was not available in a German version.

The aim of the present study was to develop and validate a German version of the STICSA. To this end, the STICSA was translated into German and assessed in two independent samples (online and in the lab). We expected to replicate the two-factorial structure of the questionnaire. We examined associations with other scales assessing anxiety, as well as depressive symptoms and stress, to establish discriminant validity and parse different components of anxiety and depression. We expected that the STICSA would be positively associated with depressive symptoms, anxiety and stress. We also expected the STICSA to better distinguish between anxiety and depressive symptoms, possibly with the somatic subscale being less influential in the dynamic interactions between anxious and depressive symptoms.

## Materials and Method

### Samples

#### Sample Size Estimation

Minimum sample size for factor analysis was estimated based on simulation studies by Gagne and Hancock (2006), who proposed a method that bases sample size estimation on measurement model quality or reliability, which can both be derived from the number of indicators per factor and the factor loadings of each indicator. Therefore, taking into account the number of indicators per factor (*n* = 10 and *n* = 11, respectively) and the factor loadings of the original questionnaire, we estimated a minimum sample size of *N* = 250.

#### Sample 1

Complete data from 510 individuals were collected online using the internet platform LimeSurvey (LimeSurvey Project Team, 2015) and participants’ identity remained anonymous to the research team. All participants were above 18 years of age and were native speakers of German. 209 participants were excluded due to either false responding to the control items (*n* = 17), no fluency in German (*n* = 7), the presence of current or past self-reported mental disorders other than anxiety disorders or depression (*n* = 95), or neurological disorders (*n* = 90). Other mental and neurological disorders were excluded to distinctly examine anxious and depressive symptoms, and avoid confounding effects (e.g. Bulloch et al., 2015). The final sample included 301 participants (mean age 26.6 years ± 8.8 standard deviation (*SD*), range 18-62 years; 67.1% female and 0.1% diverse; 96.7% had completed advanced education degrees; 19.9% self-reported diagnoses of anxiety and/or depressive disorders). Participants could take part in a lottery to win 10 Euro.

#### Sample 2

Complete data from 311 individuals were collected using the internet platform LimeSurvey (LimeSurvey Project Team, 2015) during a session in the lab as part of another research project. All participants were above 18 years of age, native speakers of German and had no neurological disorders. 8 participants were excluded due to the presence of current or past self-reported mental disorders other than anxiety disorders or depression. The final sample included 303 participants (mean age 24.9 years ± 5.2 standard deviation (*SD*), range 18-45 years; 48.8% female; 93.4% had completed advanced education degrees; 7.6% self-reported diagnoses of anxiety and/or depressive disorders). Participants were compensated for their participation with 10 Euro per hour.

The ethics committee at the Technische Universität Dresden approved all study procedures (EK 330082018) and study procedures for *Sample 2* (EK 372092017, and EK 585122019).

### Measures

The assessment for *Sample 1* included both the STICSA state and trait (Ree et al., 2008), the STAI (Laux et al., 1981; Spielberger et al., 1983), the Depression Anxiety Stress Scales (DASS-21; Henry & Crawford, 2005; Nilges & Essau, 2015), and the Beck Depression Inventory II (BDI; Beck et al., 1996; Kühner et al., 2007). For more information on these measures see the Supplementary Materials. We also obtained information about gender, age, education level, presence of mental and neurological disorders, and native language. Two control items to check for attention were included (Meade & Craig, 2012). The order of the questionnaires was randomized across participants. The assessment for *Sample 2* included the STICSA trait (Ree et al., 2008) as well as information about gender, age, education level, and native language. Bilingual psychologists translated the STICSA into German and back into English. The retranslated questionnaire was compared to the original version. Differing items were discussed and adapted.

### Data Analysis

To validate the German version of the STICSA trait, we first performed exploratory factor analysis (EFA) with oblique rotation (oblimin) and maximum likelihood estimation on *Sample 1*. Due to non-normality of the data, as assessed by Mardia’s test (Mardia, 1970), the analysis was conducted on a polychoric correlation matrix (Holgado–Tello et al., 2010). To extract the number of factors or components, we used techniques with comparably high accuracy rates (Ruscio & Roche, 2012): parallel analysis for component extraction (PA), minimum average partial procedure (MAP), optimal coordinates (OC), acceleration factor (AF) and comparison data (CD). To validate the factorial structure of the STICSA trait, we performed a confirmatory factor analysis (CFA), also based on a polychoric correlation matrix, on *Sample 2*. We used the diagonally weighted least squares (WLSMV) estimator, which is specifically designed for ordinal data (Li, 2016). Reliability was assessed using McDonald’s omega and Cronbach’s alpha (Cronbach, 1951; McDonald, 2013; Revelle & Zinbarg, 2009). Convergent and discriminant validity were examined using Kendall’s tau correlations (Kendall, 1938) with measures of individual traits that have been linked to anxiety, within *Sample 1*. Kendall’s tau has been shown to be a better estimate of the correlation in the population if the data is distributed non-normally (Howell, 2012). A validation of the STICSA state can be found within the Supplementary Materials.

To analyze the dynamic connections between the assessed traits, we used a network approach and estimated a standardized Gaussian Graphical Model (GGM) using the graphical lasso as a regularization method; the tuning parameter was selected according to the Extended Bayesian information criterion (Chen & Chen, 2008; Foygel & Drton, 2010; Friedman et al., 2008; Lauritzen, 1996). The analysis was performed based on polychoric correlations within *Sample 1* (Epskamp & Fried, 2018). Edge weight, or correlation accuracy and stability of node centrality indices as measures of node importance were assessed using bootstrapping (see Epskamp et al., 2018). An alternative model for comparison of network estimation was also estimated, see Supplementary Materials. Data and code are available at OSF (Overmeyer & Endrass, 2023a). All analyses were carried out with R (R Core Team, 2018), for used packages see Supplementary Materials.

## Results

### Exploratory Factor Analysis (Sample 1)

Assumptions for EFA were met (see Supplementary Materials). An initial analysis was conducted to extract the number of factors to retain. PA extracted two components, MAP, CD and AF extracted 2 factors and OC extracted five factors. We analyzed the data using five and two factors. Compared to the two-factor solution, the five-factor solution yielded more cross loadings and did not seem to adhere to meaningful constructs (see Supplementary Materials). Due to the more convincing results from the two-factor solution, two factors were retained in the analysis (for analysis choice recommendations see Costello & Osborne, 2005; Fabrigar et al., 1999). Table 1 displays the factor loadings after rotation. Item clustering replicated the factors from the original STICSA *cognitive* and *somatic* factors. Factors were correlated, ϕ = 0.61, 95% CI [0.50, 0.66].

**Table 1:** Oblimin Rotated Standardized Loadings (Pattern Matrix) Based Upon Polychoric Correlation Matrix

Item No.	STICSA *cognitive*	STICSA *somatic*
Item 3	**0.72**	0.17
Item 4	**0.59**	0.02
Item 5	**0.41**	0.19
Item 9	**0.80**	-0.01
Item 10	**0.87**	-0.07
Item 13	**0.76**	0.04
Item 16	**0.64**	0.01
Item 17	**0.61**	0.08
Item 19	**0.78**	-0.02
Item 11	**0.22**	0.13
Item 1	-0.01	**0.57**
Item 2	-0.15	**0.77**
Item 6	0.31	**0.49**
Item 7	0.24	**0.56**
Item 8	0.09	**0.67**
Item 12	-0.07	**0.62**
Item 14	0.08	**0.63**
Item 15	-0.01	**0.55**
Item 18	0.17	**0.69**
Item 20	0.21	**0.51**
Item 21	-0.19	**0.64**

*Note.* STICSA cognitive and STICSA somatic = State-Trait Inventory for Cognitive and Somatic Anxiety, cognitive and somatic symptoms subscales (STICSA trait).

### Confirmatory Factor Analysis (Sample 2)

As a second analysis, we performed a CFA, also on a polychoric correlation matrix. Goodness of Fit for the proposed model was tested via Root Mean Square Error of Approximation, RMSEA*_robust_* = 0.04, 95% CI [0.03, 0.05], and Tucker Lewis Index of factoring reliability (TLI*_robust_* = 0.95), values of RMSEA close to 0.06 and TLI close to 0.95 indicate acceptable fit (Hu & Bentler, 1999). Additionally, the RMSEA test of close fit (χ^2^ = 247, *df* = 188, *p* = .998) indicates close fit, and the RMSEA test of not-close fit (χ^2^ = 247, *df* = 188, *p* < .001) indicates the model does not fit poorly (MacCallum et al., 1996; Steiger, 2007). The χ^2^ test of model fit (χ^2^*_robust_* = 291, *df* = 188), however, was significant (*p_robust_* < .001), providing evidence against perfect model fit.

**Figure 1:**
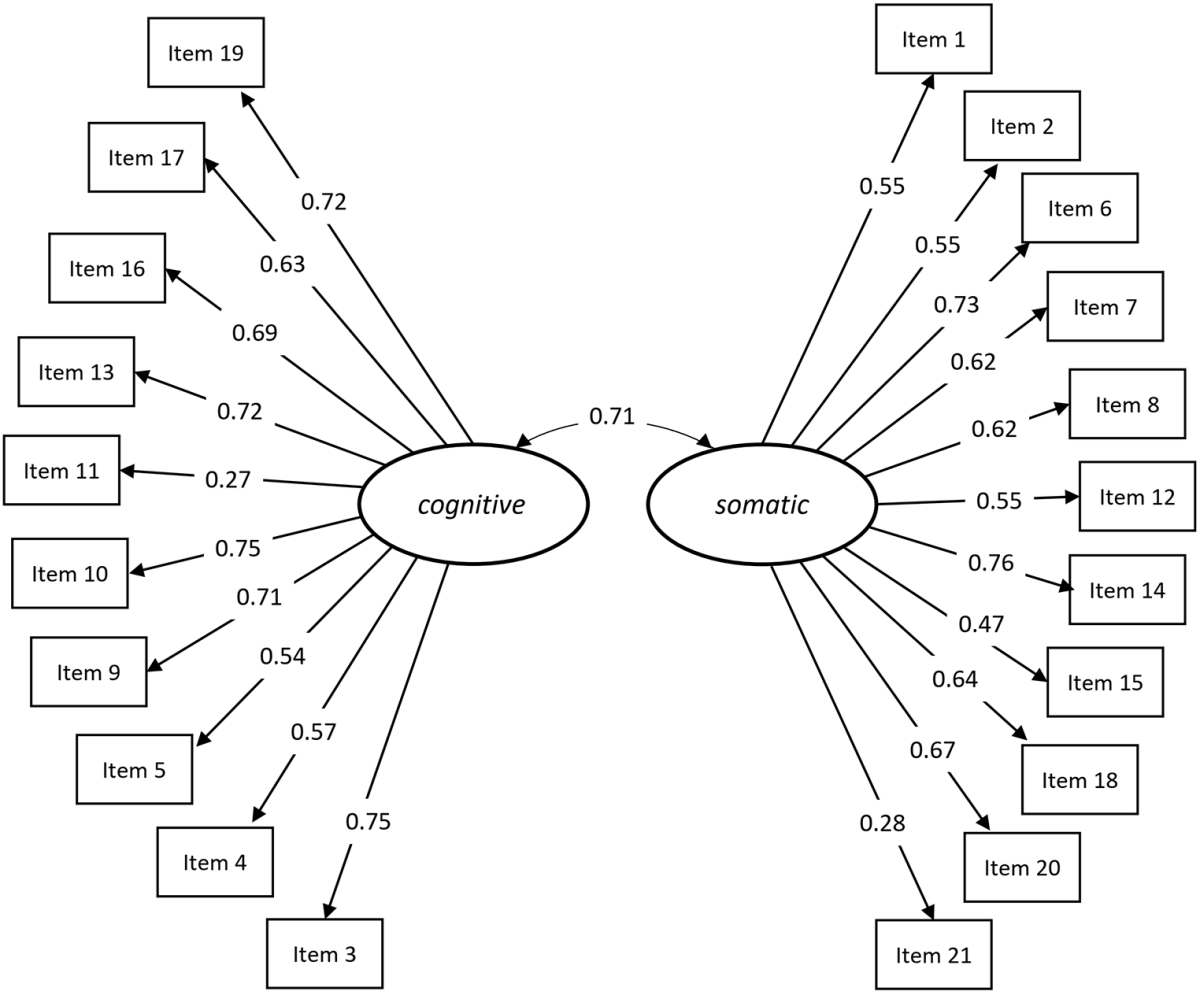
Path Diagram of the STICSA Trait (Ree et al., 2008) Results, Including All Items With Their Respective Standardized Factor Loadings on the Subscales as Well as the Correlation Between the Two Subscales

The standardized factor loadings (λ), their corresponding confidence intervals (CI) and standard errors (*SE*) are presented in Table 2. All factor loading estimates were significant and were of satisfactory magnitude. As expected, the two factors STICSA *cognitive* and *somatic* highly covaried in CFA (*cov* = 0.70; *p* < .001; 95% CI [0.61, 0.78]; *SE* = 0.04). For a visualization of the STICSA structure see Figure 1.

**Table 2:** Standardized Factor Loadings (λ) Based on Polychoric Correlations and Estimated Using Diagonally Weighted Least Squares

Item	λ	CI		*SE*
		*LL*	*UL*	
STICSA *cognitive*				
3	0.75	0.68	0.83	0.04
4	0.57	0.46	0.68	0.06
5	0.54	0.44	0.64	0.05
9	0.71	0.63	0.78	0.04
10	0.75	0.67	0.82	0.04
11	0.27	0.15	0.40	0.06
13	0.72	0.63	0.80	0.05
16	0.69	0.60	0.77	0.05
17	0.63	0.53	0.73	0.05
19	0.72	0.63	0.81	0.05
STICSA *somatic*				
1	0.55	0.44	0.66	0.05
2	0.55	0.45	0.65	0.05
6	0.73	0.62	0.85	0.04
7	0.62	0.49	0.76	0.04
8	0.62	0.50	0.75	0.04
12	0.55	0.43	0.67	0.06
14	0.76	0.61	0.91	0.06
15	0.47	0.32	0.61	0.06
18	0.64	0.51	0.61	0.04
20	0.67	0.57	0.77	0.04
21	0.28	0.15	0.42	0.07

*Note.* CI = confidence interval; *SE* = standard error; all loadings were significant. STICSA cognitive and STICSA somatic = State-Trait Inventory for Cognitive and Somatic Anxiety, cognitive and somatic symptoms subscales (STICSA trait).

### Reliability

McDonald’s omega and Cronbach’s alpha suggested satisfactory reliability for the STICSA in general (*Sample 1*: ω = 0.89, 95% CI [0.86, 0.92], α = 0.89, 95% CI [0.86, 0.91]; *Sample 2:* ω = 0.85, 95% CI [0.81, 0.88], α = 0.84, 95% CI [0.81, 0.87]), as well as for the subscales (*Sample 1*: ω_cog_ = 0.86, 95% CI [0.84, 0.89], ω_som_ = 0.81, 95% CI [0.76, 0.85], α_cog_ = 0.86, 95% CI [0.83, 0.88], α_som_ = 0.81, 95% CI [0.76, 0.85]; *Sample 2*: ω_cog_ = 0.81, 95% CI [0.77, 0.84], ω_som_ = 0.73, 95% CI [0.67, 0.78], α_cog_ = 0.81, 95% CI [0.77, 0.84], α_som_ = 0.73, 95% CI [0.67, 0.78]).

### Validity and Network Dynamics

We examined the validity of the STICSA and its subscales in *Sample 1*, see Table 3 for results. Correlations were moderate to large in magnitude. It is important to note that the tau statistic has a different metric from other correlation coefficients (see Gilpin, 1993).

**Table 3:** Kendall’s tau Correlations and Their Respective p-Value Between the Two Subscales of the STICSA and Measures of Anxiety, Depression and Stress Within Sample 1

Measure	1		2		3		4		5		6		7
	τ	*p*	τ	*p*	τ	*p*	τ	*p*	τ	*p*	τ	*p*	
1. STICSA *cognitive*	–	**–**											
2. STICSA *somatic*	**0.38**	.001	–	–									
3. STAI	**0.38**	.001	**0.24**	.001	–	–							
4. DASS *anx*	**0.44**	.001	**0.40**	.001	**0.33**	.001	–	–					
5. DASS *stress*	**0.51**	.001	**0.34**	.001	**0.32**	.001	**0.41**	.001	–	–			
6. DASS *depr*	**0.51**	.001	**0.19**	.001	**0.30**	.001	**0.31**	.001	**0.50**	.001	–	–	
7. BDI	**0.47**	.001	**0.21**	.001	**0.54**	.001	**0.37**	.001	**0.49**	.001	**0.54**	.001	–

*Note.* STICSA cognitive and STICSA somatic = State-Trait Inventory for Cognitive and Somatic Anxiety, cognitive and somatic symptoms subscale scores (STICSA trait); STAI = State-Trait Anxiety Inventory-Trait sum score; DASS anx = Depression Anxiety Stress Scales sum score of anxiety subscale; DASS stress = Depression Anxiety Stress Scales sum score of stress subscale; DASS depr = Depression Anxiety Stress Scales sum score of depression subscale; BDI = Beck Depression Inventory II sum score.

**Figure 2:**
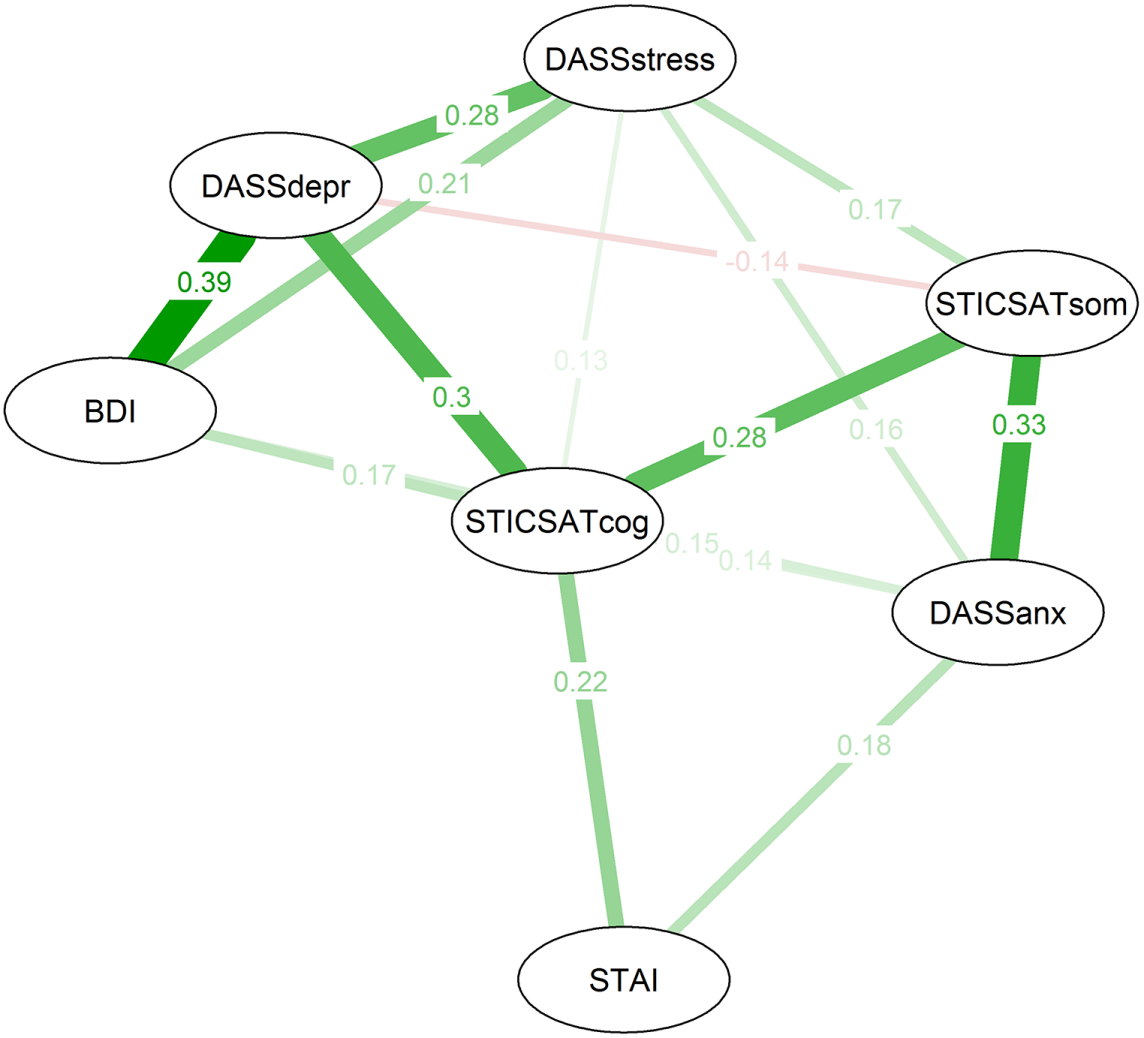
Between-Subject Graphical Lasso Network With Tuning Parameter Selected Using the Extended Bayesian Information Criterion *Note.* Nodes represent the examined self-report measures or their respective subscales for depression, stress and anxiety. Edges (connections) can be interpreted as partial correlation coefficients. Red (dashed) lines represent negative edges, green (solid) lines positive edges. STICSATcog = STICSA trait (Ree et al., 2008) cognitive subscale sum score, STICSATsom = STICSA trait (Ree et al., 2008) somatic subscale sum score, STAI = State-Trait Anxiety Inventory (STAI, Spielberger et al., 1983) sum score, DASSanx = Depression Anxiety Stress Scales (DASS-21, Henry & Crawford, 2005) anxiety subscale sum score, DASSstress = Depression Anxiety Stress Scales (DASS-21, Henry & Crawford, 2005) stress subscale sum score, DASSdepr = Depression Anxiety Stress Scales (DASS-21, Henry & Crawford, 2005) depression subscale sum score, BDI = Beck Depression Inventory II (BDI, Beck et al., 1996) sum score.

The connections between the nodes, or edge weights, within the network model calculated for Sample 1 (for a visualization see Figure 2) can be interpreted as partial correlations. They therefore represent the connection between the different measures, controlled for the presence of all other variables in the network (Borsboom & Cramer, 2013). The strongest connections were the connections between DASS anxiety and STICSA somatic (*pr* = 0.33), between STICSA somatic and STICSA cognitive (*pr* = 0.28), between BDI and DASS depression (*pr* = 0.39), between DASS depression and DASS stress (*pr* = 0.28) – and interestingly between STICSA cognitive and DASS depression (*pr* = 0.30). The connection between STICSA somatic and DASS depression was negative but small (*pr* = -0.14). STICSA cognitive appeared to be the most central node. It showed the highest values for node strength, closeness and expected influence, which indicate how strongly the node is connected to other nodes – directly as well as indirectly (Epskamp et al., 2018). The *z*-standardized raw values of centrality indices of the GGM are visualized in the Supplementary Materials. In contrast, STICSA somatic has stronger links to DASS anxiety and fewer or even negative connections with depression. Results are supported within the alternative model (see Supplementary Materials).

## Discussion

This study investigated the psychometric properties of a German version of the STICSA and dynamic associations with depressive symptoms, stress and negative affectivity. The two-factorial structure of the original version was replicated and validated for both the trait and state version of the questionnaire (see Supplementary Materials for results for the state version). All items consistently loaded on the expected factors. The somatic and cognitive anxiety factors were moderately correlated, as expected. The subscales were differentially associated with measures of anxiety and negative affectivity, depression, and stress. The cognitive subscale of the STICSA was shown to be the most central node within the network, and therefore may influence the connections between all other measures. Results show that not only is the German version of the STICSA a reliable and valid instrument, but that it also helps to distinguish the common and distinct facets of depression and anxiety.

Dynamic interactions between psychological constructs can be conceptualized within network analyses (Costantini et al., 2019). Our results suggest that cognitive symptoms, as assessed by the STICSA are at the centre of a network intertwining depressive, anxious and stress-related symptoms, with evidence that cognitive symptoms are the most influential node. Interestingly, the STAI exhibited a large correlation with the BDI, but not in the presence of other anxiety measures and stress measures. Within the network, the STAI and measures of depression only exhibited an indirect connection, with the connecting node being the cognitive symptoms of the STICSA. This fits well with research suggesting that anxiety and depressive symptoms can be differentiated using the BDI and the Beck anxiety inventory (Beck et al., 1988), particularly using items of the cognitive domain in depression and those from the physical domain in anxiety (Lee et al., 2018). A study using questionnaires as well as ecological momentary assessment found that overlapping symptoms between depression and generalized anxiety disorder bridged other symptoms across the diagnostic boundary, while cognitive and somatic symptoms still more strongly clustered within disorders (Shin, 2020). Another study identified “worrying about past” and “worrying about future” as the most prominent symptoms connecting individual depression and anxiety symptoms and “feeling unhappy” and “feeling lonely” as the most prominent disorder bridging symptoms among depression symptoms, with associations possibly explaining comorbidities (Konac et al., 2021). When integrating the approach of worry symptoms bridging disorders with the tripartite model, the finding that the cognitive symptom of worrying links depression and anxiety seems fitting: as rumination increases, the association between anxious and depressed mood is strengthened (Starr & Davila, 2012b). The insufficient focus on differences in content between anxiety and depression within the tripartite model has been criticized before (Eysenck & Fajkowska, 2018), as has the failure of the different versions of the classification systems to delineate the blurred (diagnostic) line between anxiety and depression: Demyttenaere and Heirman (2020) proposed a more phenomenological or psychopathological approach to better understand the differences between expressions of anxiety and depression. It has been suggested that the negative affectivity component can be subdivided into “worry or apprehension anxiety” and “dysthymia or valence depression” (Eysenck & Fajkowska, 2018; Fajkowska et al., 2018; Renner et al., 2018). Interestingly, there is evidence the arousal or somatic symptoms component most strongly relates to fear as measured by the Positive and Negative Affective Schedule and that the reactive and regulative functions of affect are related to the structure and function of anxiety and depression components (Domaradzka & Fajkowska, 2018). This may also explain the central role of the cognitive subscale of the STICSA within our analysis – most of the items are focused on general cognitive aspects and the subscale does not differentiate between aspects of worry vs. dysthymia.

Within the network model, the somatic subscale was only indirectly associated with the BDI, and was even negatively associated with the DASS depression subscale. These findings align with previous research indicating that the somatic anxiety subscale was less correlated with measures of depression (Tindall et al., 2021). Another study found that the somatic subscale was related to differences in both subjective and psychophysiological responses to emotional stimuli between groups of high vs. low anxiety (Barros et al., 2022). Thus, the somatic subscale of the STICSA may be useful in differentiating between anxiety and depression. However, it is essential to continuously evaluate the STICSA for future conceptualizations of anxiety. Especially research on dynamic interactions between anxiety and depression, indicating that symptoms reinforce each other, potentially explaining the high levels of comorbidity (McElroy et al., 2018), and that anxiety can worsen the severity of depression in late-life (An et al., 2019). Future research into the delineation of depression and anxiety may benefit from examining these interactions.

Limitations of the current study include the relatively small sample sizes and the high homogeneity of the samples pertaining education. Not all items may be optimal for the subscales. For Items 1, 7, 8 and 14 the highest step of the Likert scale was not used. Additionally, Items 11 and 21 showed low factor loadings (λ ≈ 0.30) on their respective subscales, and it may be discussed if it is statistically meaningful to include these items (Tabachnick et al., 2007). While the STICSA appears to clearly distinguish between cognitive and somatic aspects of anxiety, and acknowledges the multidimensionality of anxiety, it does not assess the behavioral dimension of anxiety as described by Elwood et al. (2012). This might prove an oversight, as anxiety is often marked by fearful avoidance, which may be useful as a discriminant symptom – however, it has been shown that the presence of depressive symptoms exacerbates fearful avoidance behavior (Seekatz et al., 2016). Also, cultural context might change the importance of somatic symptoms in the interaction between anxiety and depression (Escovar et al., 2018; Kim et al., 2019; Park & Kim, 2020). Despite the compelling findings on discriminant validity, there has been a study that reported evidence that the cognitive and somatic scales of the STICSA are not equally robust, with the authors concluding that the items appear to measure a mixture of both latent cognitive and somatic anxiety (Styck et al., 2022). However, Styck et al. (2022) did assess the presence of mental or neurological disorders which could influence responses for somatic symptoms (Bulloch et al., 2015) – future studies should evaluate the STICSA scales in other disorders.

### Conclusion

The German version of the STICSA appears to be a reliable and valid measure of trait and state anxiety, providing the ability to discriminate between the subscales of somatic and cognitive anxiety. As the subscales assess different facets of anxiety, it is not surprising they appear to differ in their discriminant validity and their associations to depressive symptoms and stress. Somatic symptoms of anxiety appear to most reliably assess symptoms primarily associated with anxiety, whereas cognitive symptoms seem to link anxious and depressive symptoms. The central role of cognitive symptoms in these dynamic interactions suggests that differential diagnostics should focus more on anxious somatic symptoms than on cognitive symptoms. Information gathered using the STICSA could be useful in differential diagnosis of mood and anxiety disorders, and additional understanding of both cognitive and somatic aspects of anxiety might prove useful for therapeutic interventions.

## Supplementary Materials

The Supplementary Materials for this article contain the following items (for access see Index of Supplementary Materials below):

1. The data that support the findings of this study
2. Additional information on the analysis of the STICSA trait:
  - on methods
  - on the exploratory factor analysis, with alternative factor solutions
  - on the network analysis
3. Additional information on the analysis of the STICSA state:
  - on methods
  - on the exploratory factor analysis, with alternative factor solutions
  - on the confirmatory factor analysis
4. The German Version of the STICSA trait and STICSA state

OvermeyerR.
EndrassT.
 (2023a). Differentiating anxiety and depression using a German version of the State-Trait Inventory for Cognitive and Somatic Anxiety (STICSA)
[Research data and code]. PsychOpen. 10.17605/OSF.IO/J48RG
PMC1050825537732152

OvermeyerR.
EndrassT.
 (2023b). Supplementary materials to "Cognitive symptoms link anxiety and depression within a validation of the German State-Trait Inventory for Cognitive and Somatic Anxiety (STICSA)"
[Additional information]. PsychOpen. 10.23668/psycharchives.12910
PMC1050825537732152

## Data Availability

The data that support the findings of this study are openly available at the Open Science Framework (OSF) (Overmeyer & Endrass, 2023a).
